# The Impact of the Rapid Blood Culture Identification Panel on Antibiotic Treatment and Clinical Outcomes in Bloodstream Infections, Particularly Those Associated with Multidrug-Resistant Micro-Organisms

**DOI:** 10.3390/diagnostics13233504

**Published:** 2023-11-22

**Authors:** Ji-Yun Bae, Jiyeon Bae, Min-Kyung So, Hee-Jung Choi, Miae Lee

**Affiliations:** 1 Department of Internal Medicine, Ewha Womans University College of Medicine, Seoul 07985, Republic of Korea; jiyunbae@gmail.com (J.-Y.B.); jiyeonbae89@naver.com (J.B.); heechoi@ewha.ac.kr (H.-J.C.); 2 Department of Laboratory Medicine, Ewha Womans University College of Medicine, Seoul 07985, Republic of Korea; mkso79@gmail.com; 3 Department of Laboratory Medicine, Seegene Medical Foundation, Seoul 04805, Republic of Korea

**Keywords:** blood culture, multiplex polymerase chain reaction, antimicrobial drug resistance, bacteremia

## Abstract

We evaluated the impact of the FilmArray blood culture identification (BCID) panel on the time taken to administer effective antibiotics and the clinical outcomes of bloodstream infections. We retrospectively screened patients with bloodstream infections who underwent BCID testing and compared them to a historical control group that received conventional culture testing. A total of 144 and 214 patients who underwent BCID and conventional cultures, respectively, were compared. The 30-day mortality (BCID: 9.7% vs. conventional method: 10.7%, *p* = 0.755), time to effective antibiotic administration (3 h for both BCID and conventional method, *p* = 0.789), and time to appropriate antibiotic administration did not differ significantly between the groups. BCID was not significantly associated with 30-day mortality after adjusting for the Pitt bacteremia score and the Charlson comorbidity index (adjusted OR = 0.833, CI; 0.398–1.743). Compared with conventional methods, BCID reduced the time to administration of effective antibiotics in cases of carbapenem-resistant *Enterobacterales* (CRE) (39 h vs. 93 h, *p* = 0.012) and vancomycin-resistant enterococci (VRE) (50 h vs. 92 h, *p* < 0.001) bacteremia. BCID did not affect the clinical outcomes of overall bloodstream infections; however, it contributed to the early administration of effective antibiotics in cases of CRE and VRE bacteremia.

## 1. Introduction

Bacteremia and sepsis are among the most devastating infectious diseases, associated with high mortality and complications [1]. Furthermore, the increase in multidrug-resistant micro-organisms makes the treatment of bacteremia even more challenging [2]. Timely administration of effective antibiotics is crucial in improving the outcome of bacteremia [3,4]. Therefore, rapid identification of causative organisms and detection of lead resistance determinants is important for targeted antimicrobial therapy and optimal patient management [5]. Numerous novel technologies such as rapid molecular assays and mass spectrometry have been introduced to speed up the processing of positive blood cultures [6,7,8,9].

The FilmArray blood culture identification (BCID) panel (BioFire Diagnostics, Salt Lake City, UT, USA) is a multiplexed polymerase chain reaction (PCR)-based diagnostic test that has received approval for use in positive blood cultures [10]. This test can detect 19 bacteria (*Staphylococcus* spp., *S. aureus*, *Streptococcus* spp., *S. agalactiae*, *S. pyogenes*, *S. pneumoniae*, *Enterococcus* spp., *Listeria monocytogenes*, *Enterobacteriaceae*, *Escherichia coli*, *Enterobacter cloacae* complex, *Klebsiella oxytoca*, *K. pneumoniae*, *Serratia* spp., *Proteus* spp., *Acinetobacter baumannii*, *Haemophilus influenzae*, *Neisseria meningitidis*, and *Pseudomonas aeruginosa*), five *Candida* spp. (*Candida albicans*, *C. glabrata*, *C. krusei*, *C. parapsilosis*, and *C. tropicalis*) and three antimicrobial resistance determinants (*mecA*, *vanA/B*, and *bla*_KPC_) directly from blood culture specimens. The results can be obtained within approximately 1 h from the time a positive culture is detected [11]. The high concordance of BCID with conventional culture methods has been established by many studies [12,13]. Since BCID detects antimicrobial resistance genes, it allows for the early recognition of carbapenem-resistant *Enterobacterales* (CRE) and vancomycin-resistant enterococci (VRE) bacteremia. Notably, many studies have reported that BCID enables earlier identification of bacterial species than conventional methods [7,13,14].

The appropriate interpretation of the panel results is required to determine the appropriate antibiotics for administration. Therefore, BCID tests do not necessarily guarantee the early administration of appropriate antibiotics or improved patient outcomes [15,16]. Moreover, as BCID increases the overall cost of testing, it is necessary to investigate whether it improves the clinical outcomes of patients with bacteremia [7]. Several studies have reported a shortened time to the administration of effective antibiotics when BCID is used [7,14,17]. However, the role of BCID in improving clinical outcomes, such as mortality and length of hospital stay, remains controversial [7,14,17,18].

We hypothesized that heterogeneous characteristics of the study participants may obscure the effect of BCID on clinical outcomes. Therefore, in addition to investigating its effectiveness in managing overall bloodstream infections, we analyzed its usefulness in specific patient groups that may be vulnerable to delays in treatment and cases with CRE, carbapenem-resistant *Acinetobacter baumannii* (CRAB), and VRE bacteremia.

In this study, we aimed to investigate the clinical usefulness of BCID in managing overall bloodstream infections, focusing on the early administration of effective antibiotics and examining its impact on patients’ clinical outcomes. Additionally, we sought to investigate its effects in cases of CRE, CRAB, and VRE bacteremia.

## 2. Materials and Methods

### 2.1. Study Design and Settings

This retrospective study was conducted in a university hospital with 700 beds in the Republic of Korea. This hospital has 5 adult intensive care units (one medical, one cardiac, one surgical, one neurological, and one emergency), one neonate intensive care unit, a sub-intensive care unit, a stroke unit, a hematopoietic stem cell transplantation unit, a rehabilitation treatment room, a delivery room, a dialysis unit, hematology wards, isolation wards, an emergency medical center, and operating rooms. This institution implemented BCID testing for blood cultures in September 2019. Therefore, we screened patients with bacteremia before and after the implementation of BCID.

To investigate the impact of BCID on overall bloodstream infections, patients with bacteremia who were tested using BCID were screened between January 2021 and June 2021. For controls, we identified patients with bacteremia who underwent blood culture identification and susceptibility tests using conventional methods between January 2019 and June 2019. Patients with polymicrobial bloodstream infections, and those with common skin contaminants isolated from only one pair of blood culture samples, were excluded. If these were isolated from 2 or more pairs of blood culture samples, they were considered to constitute true pathogens and were included in the analysis. Patients who were either discharged or deceased within two days were excluded from the data set since BCID could be conducted only on patients who were hospitalized until the positive culture results were reported.

In addition, we performed subgroup analysis to demonstrate the patient group that benefitted the most from BCID. The subgroups include patients in intensive care units (ICU), patients admitted to the hematology department, patients with hospital-acquired infections, and patients with Gram-positive and Gram-negative bacteremia.

To investigate the impact of BCID on bloodstream infections caused by drug-resistant micro-organisms, we screened patients with CRE, CRAB, and VRE bacteremia who underwent BCID testing between October 2019 and December 2022. As controls, we identified patients with CRE, CRAB, and VRE bacteremia who underwent blood culture identification and susceptibility tests, performed using conventional methods, between January 2018 and April 2019. Furthermore, patients who were discharged or died within 2 days of bacteremia and those with polymicrobial bloodstream infections were excluded.

### 2.2. Conventional Identification and Antimicrobial Susceptibility Methods

Both aerobic and anaerobic blood culture bottles were collected. The blood samples were cultured using the BACT/ALERT VIRTUO automated blood culture system (bioMérieux, Marcy l’Etoile, France), following the manufacturer’s instructions. Micro-organisms grown on agar plates were primarily identified using VITEK MS MALDI-TOF mass spectrometry (bioMérieux, Marcy l’Etoile, France) or the VITEK2 identification system (bioMérieux, Marcy l’Etoile, France). Results were confirmed using the various tests described in the previous study [13].

The conventional antimicrobial susceptibility testing (AST) was performed using a VITEK2 susceptibility system (bioMérieux, Marcy l’Etoile, France), and categorizations (susceptible, intermediate, or resistant) were made according to the Clinical and Laboratory Standards Institute (CLSI) guidelines. Additionally, the disk diffusion method was used to detect VRE and imipenem resistance in *Proteus* spp. When CRE were detected, the modified Hodge test and RAPIDEC^®^ Carba NP test (bioMérieux, Marcy l’Etoile, France) were performed according to the CLSI guidelines [19,20,21,22]. Additionally, the Xpert Carba-R assay (Cepheid, Sunnyvale, CA, USA) was performed to identify the carbapenemase genes. The Xpert Carba-R assay (Cepheid, Sunnyvale, CA, USA) tests the presence of five common carbapenemase genes—*bla*_KPC_, *bla*_NDM_, *bla*_VIM_, *bla*_IMP-1_, and *bla*_OXA-48_—according to the manufacturer’s instructions.

### 2.3. BCID Testing Process

**Figure 1:**
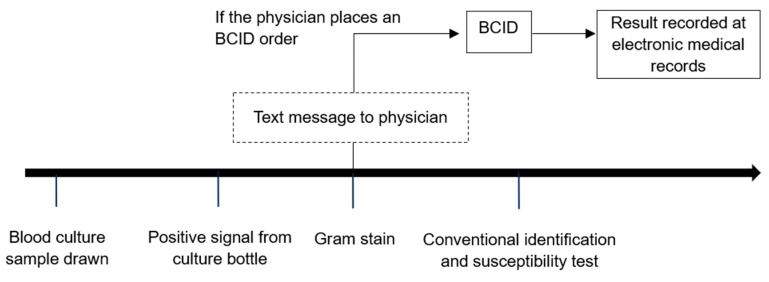
Process of conventional culture and BCID testing.

When a positive signal was detected in a blood culture bottle, Gram staining was performed, and the results were reported to the physician within 1–2 working hours using text messages to convey the option to proceed to BCID test. Simultaneously, the result was recorded in the electronic medical records. At this stage, the attending physician decided whether to perform BCID testing, given the knowledge of the positive blood culture results. If the attending physician opted for BCID testing and placed an order, the culture-positive samples were processed for BCID analysis in addition to conventional testing methods (Figure 1). Therefore, BCID panel testing was performed only for samples that had a BCID order. Although not mandatory, BCID testing for bloodstream infections was generally encouraged in our hospital due to its advantage in early bacterial species identification.

BCID testing was carried out during standard working hours (8 a.m.–5 p.m.) from Monday morning to Saturday noon. Outside of regular working hours, blood cultures were incubated until working hours resumed. If multiple positive culture samples were obtained from the same patient, BCID testing was carried out on the first positive signal.

BCID test results were recorded in the electronic medical record system. It was at the discretion of the attending physician whether to interpret the BCID results on their own or to consult with an infectious disease specialist before prescribing appropriate antibiotics.

### 2.4. BCID Panel Testing Method

When a positive signal was confirmed in the blood culture bottle and a clinician placed a BCID order, 100 μL of the positive culture medium was mixed with 500 μL of sample buffer. Then, 300 μL of this sample solution was injected into the BCID pouch. The subsequent steps, including extraction, amplification, detection, and analysis, were fully automated using a BioFire FilmArray instrument (BioFire Diagnostics, Salt Lake City, UT, USA). Each pouch contained two internal running controls, and if either control failed the result was reported as “invalid”.

### 2.5. Outcomes and Variables

The outcomes assessed were time to effective or appropriate antibiotic administration, length of hospital stay, and 30-day in-hospital mortality. Effective antibiotics were defined as those to which the corresponding organism demonstrated or implied susceptibility based on in vitro susceptibility results, with intermediate results considered ineffective. Appropriate antibiotics were defined as effective antibiotics that were adequately de-escalated.

Furthermore, time to effective or appropriate antibiotic treatment was defined as the interval between the arrival of the blood culture sample at the diagnostic laboratory and the first administration of effective or appropriate antibiotics. The length of hospital stay was defined as the number of days between the date of the first positive blood culture and the date of hospital discharge. A comparison of the length of hospital stay was analyzed only for surviving patients. Thirty-day in-hospital mortality comprised all-cause mortality. Finally, the Pitt bacteremia score and the Charlson comorbidity index were investigated because they signal known risk factors for poor outcomes in sepsis [23,24,25].

### 2.6. Statistical Analysis

The significance of differences was assessed using the Mann–Whitney U test for continuous variables and the chi-squared or Fisher’s exact test for categorical variables. A multivariate analysis was performed using logistic regression. *p*-values < 0.05 were considered statistically significant. Data were analyzed using IBM SPSS Statistics for Windows (version 25.0, IBM Corp., Armonk, NY, USA).

## 3. Results

**Figure 2:**
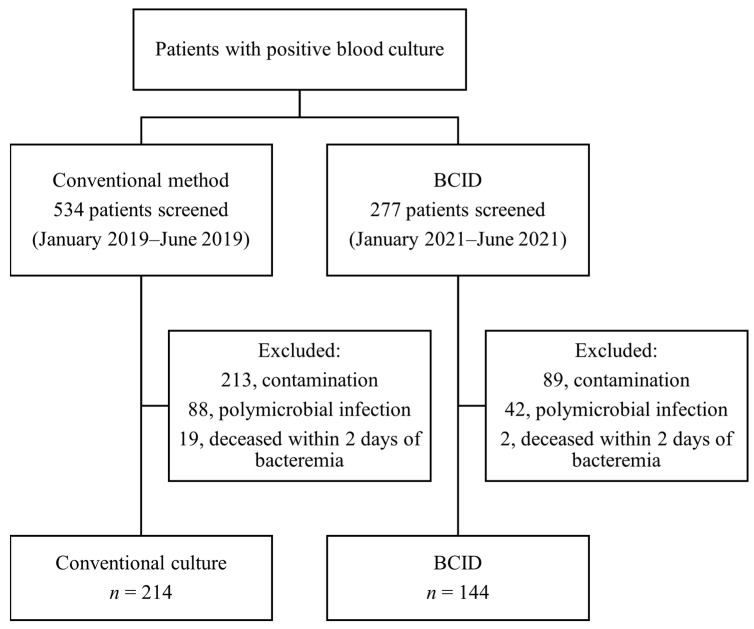
Patient enrollment flowchart.

A total of 277 patients in the BCID group and 534 patients in the conventional culture group were screened for positive blood culture results. After excluding cases of polymicrobial infection and contamination, 146 and 233 patients were included in the BCID and conventional culture groups, respectively. After excluding patients who were deceased within two days of developing bacteremia, 144 and 214 patients in the BCID and conventional culture groups, respectively, were included in the analysis (Figure 2). The baseline characteristics of the two groups are presented in Table 1. Age, Pitt bacteremia score, and Charlson comorbidity index were comparable between the groups, whereas a higher proportion of patients in ICU was noted in the BCID group than in the conventional culture group. Drug-resistant micro-organisms, such as methicillin-resistant *Staphylococcus aureus* (MRSA), VRE, CRE, and CRAB, constituted very small proportions of both groups, with no statistical differences between them. The details of micro-organisms isolated from each group are presented in Table 2. The species names are based on the final culture results. In the BCID group, 135 out of 144 cases (93.8%) involved micro-organisms detectable using the BCID panel.

The median time to the administration of effective antibiotics was 3 h in both the BCID and conventional culture groups (*p* = 0.789). The median times to administration of appropriate antibiotics were 37 and 44 h in the BCID and conventional method groups, respectively, with no statistically significant differences observed between the groups (*p* = 0.727).

Furthermore, 30-day mortality (BCID: 9.7% vs. conventional culture: 10.7%, *p* = 0.755) and length of hospital stay (BCID: 15 days vs. conventional culture: 14 days, *p* = 0.504) did not demonstrate significant difference between the groups (Table 1).

In univariable analysis, BCID was not significantly associated with 30-day mortality (odds ratio [OR] = 0.894, 95% confidence interval [CI]; 0.444–1.802), whereas the Pitt bacteremia score (OR = 1.263, 95% CI; 1.104–1.445), the Charlson comorbidity index (OR = 1.279, 95% CI; 1.122–1.458), ICU stay (OR = 2.600, 95% CI; 1.306–5.175), hematology department patients (OR = 2.778, 95% CI; 1.163–6.635), and hospital-acquired infections (OR = 2.987, 95% CI; 1.494–5.969) demonstrated significant association with 30-day mortality.

Further, BCID was included in the multivariable analysis, along with the Pitt bacteremia score and the Charlson comorbidity index. Consequently, after adjusting for these factors, BCID did not exhibit a decreased risk of 30-day mortality compared with that of the conventional culture method (adjusted OR = 0.833, 95% CI; 0.398–1.743) (Table 3).

Moreover, we performed a subgroup analysis to identify the patient group that benefited the most from BCID. However, BCID did not reduce the 30-day mortality risk in any of the subgroups studied (Table 4).

We screened 15 and 7 patients with CRE bacteremia, 52 and 34 with CRAB bacteremia, and 40 and 41 with VRE bacteremia in the BCID and conventional culture groups, respectively. Among the 15 CRE bacteremia cases in the BCID group, 12 cases were positive for *bla*_KPC_ according to the BCID test. The remaining three CRE cases missed by BCID were one NDM producer and two non-carbapenemase-producing CRE according to the Xpert Carba-R assay (Cepheid, Sunnyvale, CA, USA). The *vanA/B* gene was detected in all 40 cases of VRE bacteremia in the BCID group. The adoption of BCID testing significantly reduced the time to effective antibiotic administration in cases of CRE (BCID: 39 h vs. conventional method: 93 h, *p* = 0.012) and VRE (BCID: 50 h vs. conventional method: 92 h, *p* < 0.001) bacteremia, but not in cases of CRAB bacteremia. However, 30-day in-hospital mortality was not reduced when adopting BCID testing in any of the CRE, CRAB, or VRE bacteremia groups (Table 5).

## 4. Discussion

In the present study, we investigated the effects of BCID testing on patient outcomes and the time to effective antibiotic administration in patients with bacteremia. Our findings did not reveal a significant improvement in clinical outcomes, including 30-day mortality and length of hospital stay, when BCID was implemented. Similarly, the time to effective/appropriate antibiotic administration was not significantly shortened upon the adoption of BCID testing. However, when bacteremia with multidrug-resistant organisms was analyzed separately, the time to effective antibiotic administration was significantly reduced by BCID in cases of CRE and VRE bacteremia.

The median time to effective antibiotic administration was 3 h for both BCID testing and conventional methods, with no significant differences between them, suggesting that clinicians tend to choose empirical antibiotics that have broad-spectrum coverage against possible micro-organisms as the initial therapy. Notably, there was a very small proportion of drug-resistant micro-organisms, such as VRE and CRE, in both groups. Therefore, the effects of early identification of these micro-organisms hardly affected the overall reduction in time to effective antibiotic administration. Moreover, no significant difference in the time to appropriate antibiotic administration was observed between BCID testing and conventional methods. Regarding the fact that the time between the blood culture reception and the BCID result reporting was 29.89 h in our institution, there were time lags between the BCID reporting and appropriate de-escalation [13]. This could be attributed to two possible factors. First, the BCID panel provides limited information on antimicrobial susceptibility, primarily detecting three resistance genes. In particular, BCID testing does not determine whether a micro-organism produces expanded-spectrum β-lactamase, thereby limiting its usefulness in antibiotic de-escalation. These limitations have been partially addressed in the updated version of the panel (BCID2) [26,27,28,29]. Second, the antimicrobial stewardship program in our hospital consisted of preauthorization and did not include real-time audit and feedback for all positive blood culture results. Because BCID testing was not paired with real-time review and intervention in this study, the results of the testing might not have been interpreted well, potentially diminishing its overall usefulness [30,31]. According to the previous reports, the misinterpretation rate of BCID results was as high as 50% [31]. The Infectious Diseases Society of America recommends the implementation of rapid diagnostic tests with adequate antimicrobial stewardship programs [32].

The lack of significant reduction in mortality within the BCID group is in line with the findings of previous studies [7,14,17,33]. Notably, even a prospective randomized controlled trial failed to demonstrate any improvement in 30-day mortality, readmission rate, or length of stay [7]. Although this was a retrospective observational study and the clinical outcome of bloodstream infections could have been affected by infection severity and patient comorbidities, we adjusted for these effects using the Pitt bacteremia score and the Charlson comorbidity index.

Since many previous studies have not established a mortality benefit for BCID testing in overall bloodstream infections, we sought to identify specific patient groups that might derive the greatest benefits from BCID testing, assuming that the outcome of bloodstream infections in critically ill or immunocompromised patients may be affected by the timely administration of effective antibiotics [34,35]. Additionally, we assumed that hospital-acquired infections, in which the proportion of multidrug-resistant organisms is relatively high, might also be affected by timely treatment. However, our study did not find evidence supporting the effectiveness of BCID testing in improving patient mortality in any of the subgroups.

These results suggested that although molecular diagnostic methods are rapidly advancing to provide rapid identification of bacterial species, the clinical impact of new diagnostic tools should be thoroughly evaluated, especially using real-world data. When discrepancies emerge between the theoretical advantages and real-world usefulness of these tools, efforts should be made to identify the underlying reasons for these disparities and develop potential solutions. Since real-time audit and feedback is important, our institution is planning to implement a reporting system in which BCID results are communicated to both the attending physician and the ID physician. Another suggestion is the development of clinical decision support tools providing guidance for the interpretation of BCID.

Notably, BCID testing does not provide information on antimicrobial susceptibility of *A. baumannii.* However, in our hospital, the rate of carbapenem resistance among *A. baumannii* strains was very high (91.6%). Therefore, when *A. baumannii* was identified using BCID, it was appropriate to empirically administer antibiotics targeting CRAB before susceptibility results were reported. However, contrary to expectations, no significant differences in timely treatment were observed between the groups. First, this suggested that antimicrobial stewardship is still needed for interpreting test results and guiding the administration of appropriate antibiotics. Second, the presence of the patients under treatment for CRAB, isolated from the respiratory specimen before the development of bacteremia, might have obscured the difference in timely treatment between the groups.

Identifying VRE bacteremia was more intuitive because *vanA/B* positivity in the BCID panel indicates the presence of VRE. The concordance rate of BCID and conventional AST (VITEK2 susceptibility system and disk diffusion method) for VRE was previously reported to be 97.2% in our institution [13]. As expected, BCID testing contributed to the timely administration of linezolid in cases of VRE bacteremia. Similarly, KPC gene positivity in the BCID panel indicated the presence of carbapenemase-producing *Enterobacterales* (CPE). In our study, most cases of CRE bacteremia in the BCID group were KPC-positive, allowing for their early detection through BCID testing. The results of our study supported the usefulness of BCID testing in cases of CRE bacteremia, as early administration of effective antibiotics was achieved in the BCID group. Although mortality did not differ between the groups, patients with multidrug-resistant bacteremia may potentially benefit the most from BCID testing.

This study had several limitations. First, infection sites and adequate infection source control were not considered in the analysis of clinical outcomes [36]. Second, cost-effectiveness was not assessed, which is also an important factor when implementing new diagnostic tools. Third, MRSA was not included in the analysis of multidrug-resistant organisms, even though the *mecA* gene is included in the BCID panel. Physicians in this institution tended to prescribe vancomycin empirically when Gram-positive bacteremia is reported. Therefore, early detection of *mecA* was not expected to shorten the time to effective antibiotic administration for MRSA. We intended to select the multidrug-resistant organisms that were seldom treated empirically. Fourth, because BCID was not performed 24 h a day, 7 days a week, turnaround time might be affected by the timing of the blood culture sampling, which can affect the outcome variables. Fifth, real-time audit and feedback was not paired with BCID due to insufficient hospital resources. The decision to consult infectious disease experts was left at the discretion of the treating clinicians. Therefore, adequate interpretation of the BCID results and timely consultation with infectious disease experts might have influenced the administration of effective antibiotics and patient outcomes [37]. Nonetheless, our results may be helpful to hospitals that have adopted similar antimicrobial stewardship strategies and BCID testing protocols.

In conclusion, BCID did not improve the overall clinical outcomes in cases of bloodstream infection. However, it contributed to the early administration of effective antibiotics in cases of CRE and VRE bacteremia, suggesting that BCID testing may be useful in patients suspected of being infected with drug-resistant micro-organisms.

## Figures and Tables

**Table 1:** Baseline characteristics and clinical outcomes of the BCID and conventional culture groups.

	BCID (*n* = 144)	Conventional (*n* = 214)	*p*
Age, mean (SD)	68 (16)	68 (16)	0.932
Male sex, *n* (%)	65 (45.1)	104 (48.6)	0.520
ICU, *n* (%)	58 (40.3)	62 (29.0)	0.026
Hematology department, *n* (%)	13 (9.0)	24 (11.2)	0.505
Hospital-acquired, *n* (%)	39 (27.1)	80 (37.4)	0.043
Pitt bacteremia score, median (IQR)	2 (0–4)	2 (0–3)	0.306
Charlson comorbidity index, median (IQR)	2 (1–4)	2 (1–3)	0.808
Gram-positive, *n* (%)	58 (40.3)	112 (52.3)	0.025
Gram-negative, *n* (%)	84 (58.3)	97 (45.3)	0.016
Yeasts, *n* (%)	2 (1.4)	5 (2.3)	0.706
MRSA, *n* (%)	7 (4.9)	7 (3.3)	0.447
VRE, *n* (%)	5 (3.5)	9 (4.2)	0.726
CRE, *n* (%)	0 (0.0)	1 (0.5)	1.000
CRAB, *n* (%)	2 (1.4)	6 (2.8)	0.483
30-day mortality, *n* (%)	14 (9.7)	23 (10.7)	0.755
Length of stay, d, median (IQR)	15 (9–27)	14 (8–30)	0.504
Time to effective antibiotics, h, median (IQR)	3 (0–32)	3 (0–41)	0.789
Time to appropriate antibiotics, h, median (IQR)	37 (1–90)	44 (1–93)	0.727

BCID: FilmArray blood culture identification; SD: standard deviation; ICU: intensive care unit; IQR: interquartile range; MRSA: methicillin-resistant *Staphylococcus aureus*; VRE: vancomycin-resistant enterococci; CRE: carbapenem-resistant *Enterobacterales*; CRAB: carbapenem-resistant *Acinetobacter baumannii.*

**Table 2:** Micro-organisms isolated from the BCID and conventional culture groups.

	BCID (*n* = 144)	Conventional (*n* = 214)
Micro-organisms included in BCID panel, *n* (%)	135 (93.8)	194 (90.7)
Gram-positive		
*Enterococcus faecalis*	6	9
*Enterococcus faecium*	8	19
other *Enterococcus* spp.	0	2
*Staphylococcus aureus*	17	19
coagulase-negative staphylococci	16	30
*Streptococcus* spp.	5	20
Gram-negative		
*Citrobacter freundii*	0	1
*Citrobacter koseri*	0	1
*Enterobacter cloacae*	1	2
*Enterobacter aerogenes*	1	1
*Enterobacter ludwigii*	1	0
*Escherichia coli*	43	55
*Klebsiella pneumonia*	24	18
other *Klebsiella* spp.	2	2
*Proteus mirabilis*	3	0
*Salmonella* spp.	1	1
*Serratia marcescens*	1	0
*Acinetobacter baumannii*	2	6
*Pseudomonas aeruginosa*	3	3
Yeasts		
*Candida* spp.	1	5
Micro-organisms not included in BCID panel, *n* (%)	9 (6.3)	20 (9.3)
*Granulicatella adiacens*	0	1
*Corynebacterium striatum*	0	3
*Actinotignum schaalii*	1	0
*Clostridium* spp.	2	7
*Eggerthia catenaformis*	1	0
*Eubacterium* spp.	1	2
*Lactobacillus* spp.	1	0
*Acinetobacter ursingii*	1	0
*Chryseobacterium meningosepticum*	0	1
*Bacteroides* spp.	1	4
*Fusobacterium periodonticum*	0	1
*Prevotella nigrescens*	0	1
*Saccharomyces cerevisiae*	1	0

BCID: FilmArray blood culture identification.

**Table 3:** Effect of BCID on 30-day mortality in patients with bacteremia (multivariable analysis).

	Adjusted OR	95% CI	*p*
Pitt bacteremia score	1.304	1.133–1.501	<0.001
Charlson comorbidity index	1.317	1.147–1.513	<0.001
BCID	0.833	0.398–1.743	0.627

BCID: FilmArray blood culture identification; OR: odds ratio; CI: confidence interval.

**Table 4:** Subgroup analysis of the effect of BCID on 30-day mortality in patients with bacteremia (adjusted for Pitt bacteremia score and Charlson comorbidity index).

Subgroup	Adjusted OR	95% CI	*p*
ICU	1.563	0.558–4.378	0.396
Hematology department	1.718	0.200–14.788	0.622
Hospital-acquired	1.162	0.380–3.549	0.792
Gram-positive organism	1.519	0.534–4.317	0.433
Gram-negative organism	0.485	0.153–1.540	0.220

BCID: FilmArray blood culture identification; OR: odds ratio; CI: confidence interval; ICU: intensive care unit.

**Table 5:** Time to effective antibiotic administration and 30-day mortality in patients with CRE, CRAB, and VRE bacteremia.

	BCID	Conventional	*p*
CRE	*n* = 15	*n* = 7	
Time to effective antibiotics, h, median (IQR)	39 (27–53)	93 (63–98)	0.012
30-day mortality, *n* (%)	6 (40.0)	1 (14.3)	0.354
CRAB	*n* = 52	*n* = 34	
Time to effective antibiotics, h, median (IQR)	29 (16–52)	42 (6–78)	0.340
30-day mortality, *n* (%)	31 (59.6)	18 (52.9)	0.541
VRE	*n* = 40	*n* = 41	
Time to effective antibiotics, h, median (IQR)	50 (33–86)	92 (77–102)	<0.001
30-day mortality, *n* (%)	8 (20.0)	12 (29.3)	0.333

CRE: carbapenem-resistant *Enterobacterales*; CRAB: carbapenem-resistant *Acinetobacter baumannii*; VRE: vancomycin-resistant enterococci; BCID: FilmArray blood culture identification; IQR: interquartile range.

## Data Availability

The data presented in this study are available on request from the corresponding author.
